# Impact of non-restorative cavity control on proximal carious lesions of anterior primary teeth on the tooth survival and patient-centered outcomes (CEPECO 2): study protocol for a non-inferiority randomized clinical trial

**DOI:** 10.1186/s12903-021-01524-0

**Published:** 2021-03-31

**Authors:** Renata M. D. Bianchi, Aline M. Pascareli-Carlos, Isabela Floriano, Daniela P. Raggio, Mariana M. Braga, Thais Gimenez, Mariana C. Holanda, Gabriela S. da Silva, Karina H. de Natal, Tamara K. Tedesco

**Affiliations:** 1 Graduate Program in Dentistry, Ibirapuera University, Av. Interlagos 1329, São Paulo, SP 04661-100 Brazil; 2 School of Dentistry, Universidade Do Norte, Uninorte, Av. Joaquim Nabuco, Centro, Manaus, 12811355 Brazil; 3 School of Dentistry, University Center UNINOVAFAPI, Rua Vitorino Orthiges Fernandes 6123, Teresina, Brazil; 4 Department of Orthodontic and Pediatric Dentistry, School of Dentistry, University of São Paulo, Av. Prof. Lineu Prestes, São Paulo, 2221 Brazil; 5 School of Dentistry, FAESF Faculty, Rua Olemar Alves de Sousa 401, Floriano, Brazil

**Keywords:** Dental caries, Non-restorative treatment, Resin composite, Deciduous teeth

## Abstract

**Background:**

Studies have questioned the necessity of restoring cavitated carious lesion on primary teeth, once the control of biofilm is the most important factor to arrest these lesions. This randomized clinical trial aimed to compare the survival of teeth treated with a non-restorative cavity control (NRCC) compared to resin composite restorations (RCR) on proximal carious lesion in anterior primary teeth, as well as the impact of these treatments on patient-centered outcomes.

**Methods:**

A randomized clinical trial with two parallels arms (1:1) will be conducted. Children between 3 and 6 years old will be selected from the Center of Clinic Research of Pediatric Dentistry of Ibirapuera University (UNIB), a dental trailer (FOUSP) located on Educational Complex Professor Carlos Osmarinho de Lima, the Pediatric Dentistry Clinic of Santa Cecília University and from the Pediatric Dentistry Clinic of University Center UNINOVAFAPI. One hundred and forty-eight teeth will be randomly distributed in two experimental groups: (1) Selective removal of carious tissue and RCR; or (2) NRCC through cavity enlargement using a metallic sandpaper. The primary outcome will be tooth survival after 6, 12, 18 and 24 months. The duration and the cost of dental treatments will be considered for the estimation of the cost-effectiveness of the evaluated treatments. The discomfort reported by the participants will be measured after each treatment using the FIS scale. The participants’ satisfaction and perception of the parents/legal guardians will be evaluated through questionnaires. For the primary outcome, Kaplan–Meier’s survival and Long-Rank test will be used for comparison between the two groups. All the variables will be modeled by Cox regression with shared fragility. Significance will be considered at 5%.

**Discussion:**

The NRCC could be an option to manage carious lesions on proximal surfaces of primary teeth, and the approach could be well accepted by the children and parents/legal guardians.

*Trial registration* Clinicaltrials.gov registration: NCT03785730, Registered on December 18th 2018, first participant recruited 30/04/2019, https://clinicaltrials.gov/ct2/show/NCT03785730.

Ethics Reference No: 91569118.8.0000.5597.

Trial Sponsor: Universidade Ibirapuera.

The Trial was prospectively registered.

## Background

Despite the reduction in the prevalence of dental caries, especially in Pediatric Dentistry, caries is still considered a public health problem, since it affects more than 50% of pre-school and school-age children [1]. Data from previous studies also suggest that around 10 to 50% of these children have cavitated lesions on anterior teeth [2–4], which results in negative impact on their quality of life [4]. The evolution of the disease leads to loss of self-esteem, masticatory difficulty, pain, and frustration [4].

Thus, treatments that prevent the progression of these lesions and allow the maintenance of primary teeth until exfoliation while improving the patient's quality of life should be investigated. Restorative techniques are a treatment option for such cavitated lesions [5], but although the available restorative materials have improved, restorations often need replacement, mainly due the patient's failure to control caries risk factors [6].

Since biofilm control is the most critical factor for lesion arrestment, the approach of restoring primary teeth has been questioned [7]. This is corroborated by studies that show that a large part of primary teeth with untreated carious lesions exfoliate without showing any symptoms [8–10].

Therefore, the non-restorative cavity control (NRCC) has been suggested as an option for the treatment of carious lesions in primary teeth. This approach consists of an enlargement of the cavity to allow the adequate removal or disorganization of the daily biofilm through toothbrushing [11–13].

Studies found that this approach shows similar results regarding the improvement of oral-health-related quality of life after one year of treatment [12] and teeth longevity after 3.5 years of follow up [13] compared to conventional composite resin restoration and atraumatic restorative treatment. However, these data come from studies based on posterior teeth restorations [11–13]. Aesthetic is one of the main reasons for replacing restorations in anterior teeth [6], favoring patient engagement for oral care, could also be a stimulus for a frequent brushing of teeth treated with NRCC. However, the acceptability and longevity of this approach has not been compared with the conventional treatment.

This study aims to evaluate the impact of NRCC for proximal carious lesions in anterior primary teeth on teeth survival. The secondary outcomes will be cost-effectiveness and patients-centered outcomes between the two treatment options. We hypothesize that the survival of teeth treated with NRCC is non-inferior, with a non-inferiority margin of 15%, from that of teeth restored with composite resin.

## Methods/design

### Study design and ethical aspects

This non-inferiority randomized controlled clinical trial, with two parallel groups with 1:1 allocation ratio was reported according to the Standard Protocol Items for Clinical Trials (SPIRIT) and then registered on the ClinicalTrials.gov platform (NCT03785730). The protocol was approved by the Research Ethics Committee of Universidade Ibirapuera (UNIB). The other centers involved are considered co-participants (Centro Universitário do Norte—Uninorte, Centro Universitário Uninovafapi—UNINOVAFAPI, and School of Dentistry from the University of Sao Paulo—FOUSP).

Children aged 3 to 6 years with at least one proximal cavitated lesion on anterior primary teeth will be selected. Teeth will be randomly allocated to selective removal of carious tissue and resin composite restoration (RCR) or NRCC. Tooth survival after two years of follow-up will be the primary outcome and cost-effectiveness, satisfaction, and discomfort reported by participants and parents/guardians’ perception will be the secondary outcomes.

Only patients who fulfill the eligibility criteria will be included in the study after the legal guardians sign the informed consent form and the child consents to participate in the study.

### Sample selection

For sample size calculation, we used an expected survival of primary teeth with cavitated lesions in dentin affecting the proximal surface submitted to NRCC of 89.7% after 24 months [13] and a clinically significant difference of 15% in the success rate between groups. Considering a non-inferiority study, a significance level of 0.05 and a power of 0.80, we reached the final number of 102 teeth. Since each child can contribute with more than one tooth, 20% was added to this value (cluster per child), and 20% more was added due to possible sample losses. Thus, the final rounded number of 74 teeth per group was reached, resulting in a total of 148 teeth (sealedenvelope.com).

Children aged 3 to 6 years will be selected from the Clinical Pediatric Research Center—UNIB (CEPECO) (São Paulo, SP, BR), Pediatric Dentistry Clinic—Uninorte (Manaus, AM, BR), Pediatric Dentistry Clinic—UNINOVAFAPI (Teresina, PI, BR) and dental trailer (FOUSP) located on Educational Complex Professor Carlos Osmarinho de Lima (Barueri, SP, BR). The screening will be carried out under natural light with the aid of a wooden spatula. Potentially eligible children will be referred for clinical examination.

Recruitment will be taking place from April 2019 to December 2021. After allocation and treatment, the children will be followed up for 24 months. Table 1 displays the flow diagram of the clinical trial phases.

**Table 1:** Flow diagram of clinical trial’s phases in according to SPIRIT

Timepoint**	Study period						
	Enrolment	Allocation	Post-allocation				Close-out
	*−t*_*1*_	0	*t*_*1*_	*t*_*2(6 m)*_	*t*_*3(12 m)*_	*t*_*4(18 m)*_	*t*_*5(24 m)*_
*Enrolment*							
Eligibility screen	X						
Informed consent	X						
Allocation		X					
*Interventions*							
NRCC			X				
RCR			X				
*Assessments*							
Survival of tooth				X	X	X	X
Cost-effectiveness			X				X
Discomfort reported by participant			X				
Satisfaction of the participants				X			
Perception of the Parents/legal Guardians		X	X	X			

### Clinical examination

Initially, a clinical exam will be carried out in a dental office by the operators using a light reflector, mirror, and WHO probe, after prophylaxis to identify the eligible participants.

### Eligibility criteria

Children that present at least one cavitated carious lesion on the proximal surface of anterior teeth will be included.

Patients with special needs, who use orthodontic braces, and/or present systemic diseases that could influence the oral cavity will be excluded. Additionally, teeth with carious lesion that affect more than one third of the buccal and/or lingual surfaces, with previous history of dental trauma, pulp exposure, spontaneous pain, pathological mobility, abscess or fistula, teeth with restorations, developmental enamel defects or physiological mobility (exfoliation) will be excluded.

### Operator’s training

Four pediatric dentists will be trained to perform the two techniques (NRCC and resin composite restorations—RCR). The training will consist of theoretical classes and laboratory activities for three hours each.

### Sequence generation

Teeth will be randomly assigned into one of the groups considering the strata of the research center (UNIB, FOUSP, Uninorte, and UNINOVAFAPI), in 4, 6, and 8 blocks, according to the sequence obtained by an external examiner generated by the website www.sealedenvelope.com.

### Allocation concealment mechanism

The generated sequence will be distributed in sequentially numbered opaque sealed envelopes by an external researcher, and the envelopes will be opened by the dental assistant immediately before the treatment.

### Interventions

For teeth of the RCR Group, after prophylaxis and cotton roll isolation, selective removal of carious tissue will be performed, removing infected dentin from the pulp wall and with total removal of carious tissue of the surrounding walls, using curettes compatible with cavity size. The acid conditioning will be performed only in enamel for 15 s with 37% phosphoric acid gel. The adhesive system will then be actively applied for 20 s in both enamel and dentin, followed by air-drying for 5 s and light-curing for 10 s (Single Bond Universal; 3M/ESPE, St. Paul, USA). Resin composite (Z250, color B1; 3M/ESPE, St. Paul, USA) will then be placed in the cavity with the aid of a spatula of insertion and polyester matrix. Before finishing and polishing, the occlusion will be checked with carbon paper and adjusted if necessary.

**Fig. 1:**
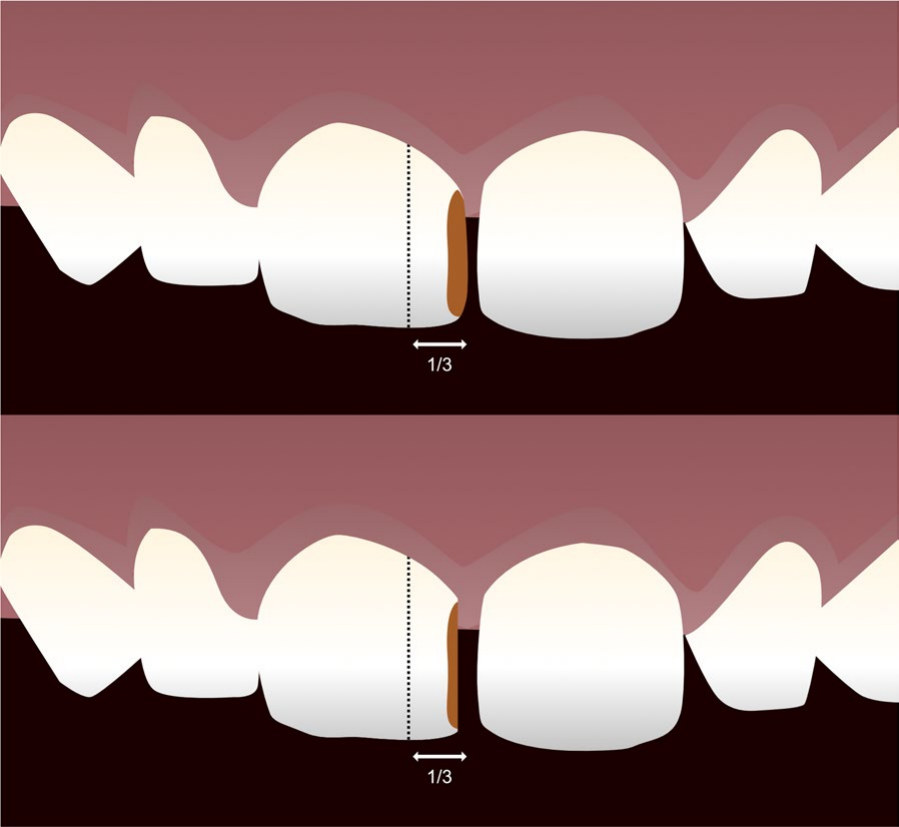
Illustration of NRCC treatment. Dashed lines represent the cavity’s size limit to be included in the study (up to one-third of the buccal and/or lingual surfaces involved in the proximal carious lesion). In the first image, we can observe a proximal carious lesion in the upper incisor. This cavity will be enlarged with metallic sandpaper in the buccal and lingual direction, exposing the cavity, as demonstrated in the second image

For teeth randomized to NRCC Group, the proximal cavities will not be restored, but enlarged with metallic sandpaper, exposing the cavity, to allow access for toothbrushing associated with 1000 ppm fluoride toothpaste [13] (Fig. 1).

All participants and their respective guardians will receive hygiene and diet instructions in the first clinical appointment and at every follow-up. Other teeth with carious lesions not included in the research will be treated according to the diagnosis. The toothbrushes and toothpaste will be also provided to the participants.

The risks for participants will be minimal and related to carious lesion progression. No adverse effect of the interventions is expected. To guarantee participant adherence to the study, social media will be used and participants will be contacted by mobile message applications.

### Blinding

Blinding of operators, participants, and examiners will not be possible due to the evident differences between the interventions. However, an external researcher not directly involved in the study will perform the statistical analysis of the data.

### Data collection and evaluated outcomes

All data obtained will be tabulated by a researcher and checked by another researcher. An identifying number will ensure participants' confidentiality. The data will be stored on the cloud with access restricted to the researchers involved in the project and the principal investigator. After completing the study and data deidentification, the data will be used in an open electronic database to ensure research transparency. Due to the short follow-up, there will be no data monitoring committee. However, the coordinator of the trial will be responsible for periodical auditing.

The explanatory variables—sex, age, toothbrushing, use of fluoride toothpaste, and dental floss—will be collected by a questionnaire with closed-ended questions. Tooth survival after two years of follow-up will be considered as the primary outcome. Secondary outcomes will be the cost-effectiveness of both treatments, satisfaction, and discomfort reported by participants and parents/guardians' perception.

Patients will be contacted by telephone for recall of the periodical appointments. For those who cannot be contacted after several phone call attempts, a letter will be sent to the home address.

Four examiners will be trained for the assessed outcomes in two stages:

1. Theoretical classes with images (three hours).
2. Clinical setting with children with similar conditions to the eligible ones, but not included in the study (three hours).

### Primary outcome

#### Tooth survival

Survival of the tooth will be evaluated at 6, 12, 18, and 24 months after the initial intervention through clinical examination. A successful treatment will be considered when the teeth is without clinical signs or symptoms of pulp pathology or exfoliated, with no need for more invasive interventions, such as pulpectomy or extraction.

In cases of failed RCR, no further treatment will be performed, and the tooth will continue to be monitored for up to 24 months. If patients report pain or present the need for more invasive interventions, the necessary treatment will be performed.

### Secondaries outcomes

#### Cost-effectiveness

An economic cost-effectiveness analysis will be carried out considering the payer perspective (Unified Health System) and tooth survival as treatment effectiveness. The direct costs for the procedures performed in each of the two groups will be measured and evaluated, as well as the labor cost of the professionals involved in the clinical attendance (direct costs). The costs of all the materials used will be computed using the average market value in different dental material stores, and these data will be updated during the study. The labor costs will be measured by the time required to perform the treatment. The time required to perform the procedure will be measured with a stopwatch. Time will start right after the prophylaxis for both groups and will be stop after the finalization of the intervention. The average time will be calculated for each group. Patient costs with transportation and time will not be analyzed (indirect costs).

Thus, the incremental cost-effectiveness ratio (ICER) will be calculated considering a rate between the total cost of each treatment and tooth survival rate after two years, according to the following formula:

$$ICER=\left[\frac{Cost\; of\; intervention\; RCR}{Survival\;\; of\; intervantion\; RCR}\right]-\left[\frac{Cost\; of\; intervention\; NRCC}{Survival\; of\; intervention \; NRCC}\right]$$

#### Discomfort reported by participants

The participant will be questioned about the discomfort related to the intervention immediately after the treatment is finished using the Face Image Scale (FIS) [14]. The scale will be shown to the children and they will be asked to point to the image that represents their discomfort level after the question: how do you feel at this moment? Without the operator’s presence, the dental assistant will ask for the participant’s most honest opinion.

#### Satisfaction of the participants

The treatment satisfaction of the participants will be rated using a close-ended questionnaire six months from the initial treatment. To each question, the participants will be asked to point the image that represents their feelings. The FIS scale will be used as explained above.

#### Perception of the parents/legal guardians

The perception of the parents or guardians concerning the treatment will be evaluated by the "Child's and parent's questionnaire about teeth appearance" [15] applied before the treatment, immediately after, and six months later. The dental assistant alone will ask for their honest opinion.

### Data analysis

The efficacy of each treatment will be evaluated according to five main outcomes:

1. Tooth survival (primary outcome): The outcome will be dichotomized as survival versus failure. Kaplan Meier’s plot with overall survival in intention-to-treat (ITT) study population will be performed. The ITT study population will include all randomized participants regardless of protocol deviations, non‐compliance, or early study withdrawal. The Log-Rank test will be used to compare the survival curves of the two groups. The multivariate Cox regression with shared frailty model will be used to evaluate the influence of the explanatory variables (sex, age, jaw, type of teeth, toothbrushing, use of fluoride toothpaste, and dental floss) on the outcome. Hazard ratios (HR) with the respective 95% confidence interval will be calculated.
2. Cost-effectiveness (secondary outcome): Incremental cost-effectiveness ratio will be calculated considering the ratio between the total cost of each treatment and tooth survival after two years.
3. Discomfort reported by the participants (secondary outcome): The data will be summarized as the mean with the standard deviation or the median with the interquartile range, as appropriate. The two groups will be compared after confirming the distribution of data (Normal/non-normal), using the Student's t-test or Mann–Whitney test.
4. Satisfaction of the participants (secondary outcome): The data will be summarized as the mean with the standard deviation or the median with the interquartile range, as appropriate. The two groups will be compared after confirming the distribution of data (Normal/non-normal), using the Student's t-test or Mann–Whitney test.
5. Perception of the Parents/Legal Guardians (secondary outcome): The data will be summarized as the frequencies. The two groups will be compared using the Chi-Square Test.

For all analyzes, the significance value will be set at 5%.

### Patient and public involvement

The research question arose from the need to seek new treatment options that prevent proximal carious lesion in front teeth to progress and enable the teeth to be naturally exfoliated. At the same time, the treatment should have a good acceptability by children and parents or guardians. Since this is a clinical trial, there will be no involvement of patients or the public in the study design.

### Ethics and dissemination plan

This randomized clinical trial was approved by the Research Ethics Committee of Universidade Ibirapuera (UNIB) (protocol number 1.670.059). The other centers involved are considered co-participants (Centro Universitário do Norte—Uninorte, Centro Universitário Uninovafapi—UNINOVAFAPI, and School of Dentistry of the University of Sao Paulo—FOUSP). The participants will be included after their parents or guardians sign the Informed Consent Form containing detailed information about the research, and the children’s verbal consent. All the data will be tabulated by a researcher and checked by another researcher. An identification number will ensure the participants' confidentiality. After data deidentification, data will be stored in an open electronic database to ensure transparency. The results of this study will be presented in conferences and published in peer-reviewed journals, as well as in social media to facilitate the science translation to lay audiences. Moreover, any required protocol modifications will be performed in Clinicaltrials.gov website.

## Discussion

Since biofilm control is the most critical factor for lesion arrestment, previous studies have questioned the approach of restoring primary teeth [8–10]. Thus, non-restorative cavity control has been suggested as a new approach for proximal carious lesion on primary teeth, which allow the disorganization of the biofilm, avoiding the progression of these lesions until exfoliation. To the best of your knowledge, this is the first randomized clinical trial (RCT) that aims to evaluate the NRCC treatment option on anterior primary teeth.

Although the use of silver diamine fluoride for carious lesion management in primary teeth could be another option, an umbrella review has reported the black staining of the arrested lesion as the most common side effect, which in anterior teeth is not well-accepted for aesthetic reasons [16].

It is important to highlight that the primary outcome of this study is tooth survival since the objective of restoring primary teeth is their maintenance until the exfoliation. However, this outcome is not commonly reported in studies that evaluate carious lesion treatments. Due to the follow-up time, not all teeth included in the study are expected to exfoliate. Thus, teeth without clinical signs or symptoms of pulp involvement, which do not require more invasive interventions, will also be considered as treatment success. The follow-up until exfoliation of all teeth, although ideal, would increase the time for completion of the RCT, increasing considerably the research cost without extra benefits concerning the outcome. Moreover, since the patient's opinion is essential for the decision-making process on evidence-based dentistry, we will also assess patient-centered outcomes.

Finally, this study aims to investigate whether a non-restorative treatment in proximal cavities of anterior primary teeth is a viable treatment option. If confirmed, our hypothesis will support the use of a non-restorative approach, which has the potential to cause less discomfort for pediatric patients, has a shorter chair-time, and a lower cost to the population.

## Data Availability

After deidentification, they will be stored in an open electronic database—Mendeley data (data.mendeley.com)—in order to sure the search transparency.

## Abbreviations

NRCC: Non-restorative cavity control
RCR: Resin composite restorations
RCT: Randomized clinical trial
